# The similarity of the structure of DSM-IV criteria for major depression in depressed women from China, the United States and Europe

**DOI:** 10.1017/S0033291714003067

**Published:** 2015-03-17

**Authors:** K. S. Kendler, S. H. Aggen, Y. Li, C. M. Lewis, G. Breen, D. I. Boomsma, M. Bot, B. W. J. H. Penninx, J. Flint

**Affiliations:** 1 Virginia Institute for Psychiatric and Behavioral Genetics, Virginia Commonwealth University, Richmond, VA, USA; 2 Department of Psychiatry, Virginia Commonwealth University, Richmond, VA, USA; 3 Department of Human and Molecular Genetics, Virginia Commonwealth University, Richmond, VA, USA; 4 Wellcome Trust Centre for Human Genetics, Oxford, UK; 5 MRC SGDP Centre, Institute of Psychiatry, King's College London, London, UK; 6 National Institute for Health Research Biomedical Research Centre for Mental Health at the Maudsley and Institute of Psychiatry, King's College London, London, UK; 7 Department of Biological Psychology and EMGO Institute of Health and Care Research, VU University, Amsterdam, The Netherlands; 8 Department of Psychiatry and EMGO Institute of Health and Care Research, VU University Medical Center, Amsterdam, The Netherlands

**Keywords:** China, cross-cultural psychiatry, DSM-IV, major depression, measurement invariance

## Abstract

**Background:**

Do DSM-IV diagnostic criteria for major depression (MD) in Chinese and Western women perform in a similar manner?

**Method:**

The CONVERGE study included interview-based assessments of women of Han Chinese descent with treated recurrent MD. Using Mplus software, we investigated the overall degree of between-sample measurement invariance (MI) for DSM-IV diagnostic criteria for MD in the CONVERGE sample and samples selected from four major Western studies from the USA and Europe matched to the inclusion criteria of CONVERGE. These analyses were performed one pair at a time. We then compared the results from CONVERGE paired with Western samples to those obtained when examining levels of MI between pairs of the Western samples.

**Results:**

Assuming a single factor model for the nine diagnostic criteria for MD, the level of MI based on global fit indexes observed between the CONVERGE and the four Western samples was very similar to that seen between the Western samples. Comparable results were obtained when using a two-factor structure for MI testing when applied to the 14 diagnostic criteria for MD disaggregated for weight, appetite, sleep, and psychomotor changes.

**Conclusions:**

Despite differences in language, ethnicity and culture, DSM criteria for MD perform similarly in Chinese women with recurrent MD and comparable subjects from the USA and Europe. The DSM criteria for MD may assess depressive symptoms that are relatively insensitive to cultural and ethnic differences. These results support efforts to compare findings from depressed patients in China and Western countries.

## Introduction

The importance of cultural factors on psychiatric illness has been a subject of considerable interest in discussions concerning major depression (MD) in the world's largest population, that of China. Cultural factors are believed to alter the experience of MD, influencing the way sufferers recognize and describe their symptoms, with implications for both diagnosis and our understanding of the nature of the illness (Kleinman, 1982, 1986, 2004; Parker *et al*. 2001). Prevalence rates of MD are typically lower in China and other East Asian countries than commonly seen elsewhere in the world (Weissman *et al.* 1996; Chen *et al.* 1999; Lee *et al.* 2009) and MD is often associated with higher levels of disability compared to Western countries (Simon *et al.* 2002). This has been ascribed to a reduced tendency to report psychiatric symptoms unless very impairing, a perspective termed ‘cultural stoicism’ (Chang *et al.* 2008; Liao *et al.* 2012).

It has been claimed that culturally coded symptoms obscure the recognition of depression when Western diagnostic criteria are applied. Indeed, applying the American Psychiatric Association's Diagnostic and Statistical Manual or DSM-IV criteria in a different cultural setting may be a ‘category fallacy’ (Kleinman, 1977). Furthermore, ignorance of the phenomenology of depression in Chinese, among whom somatization is a common manifestation (Ryder *et al.* 2008), may obscure the full range of the depressive experience and bias our understanding of its nature. This raises the important question as to the extent to which depression in China is the same as in Western societies.

Without biological markers of MD and in the face of continuing ignorance as to its biological origins, answers to this question have to come from studies of MD's known risk factors and phenomenology. In a series of studies, the China, Oxford and Virginia Commonwealth University Experimental Research on Genetic Epidemiology (CONVERGE) study of MD has carried out detailed clinical assessments of ~6000 cases of recurrent MD in Han Chinese women and ~6000 matched controls (Flint *et al.* 2012). Congruent with studies in European-derived populations (Brown & Harris, 1978; Kendler *et al*. 1998, 2000; Fergusson & Mullen, 1999; Parker, 1990), results from this study have shown that the risk for MD in Chinese women is appreciably increased by childhood sexual abuse (Cong *et al.* 2012; Chen *et al.* 2014), poor parenting (Gao *et al.* 2012), and stressful life events (Tao *et al.* 2011). Many other clinical features of MD seen in Western samples (e.g. Boyd *et al.* 1984; Schatzberg & Rothschild, 1992; Kendler *et al.* 2005) have also been replicated in the CONVERGE sample such as the associations between early age at onset of MD and a positive family history, co-morbidity with anxiety disorders and high recurrence (Yang *et al.* 2014), and the strong association between MD and the personality trait of neuroticism (Xia *et al.* 2011).

However, a key question remains: to what extent are the symptomatic manifestations of the illness similar in China and Western countries? One way to answer this question is to test the assumption that symptom profiles from these populations represent the same underlying latent construct. In a study in 15 primary-care centers across 14 countries (including China and Japan), Simon *et al.* investigated the latent structure of the nine DSM-IV (APA, 1994) criteria for MD in three groups of centers reporting low, medium and high prevalence rates for MD (Simon *et al.* 2002) (Chinese and Japanese samples were both in the low-prevalence group). They applied a categorical factor analysis of the nine DSM-IV MD criteria and when examining the first factor noted that ‘the pattern of symptom loadings on this component was remarkably similar in all three groups’ (Simon *et al.* 2002, p. 589).

Another way to look at the similarity of the MD construct is to investigate measurement invariance (MI). Within the common factor model, MI is a statistical test of the properties of items in a scale or criteria for a diagnosis that indicates the degree to which they are measuring the same underlying construct in different groups of subjects. For any two large-scale data collection projects, there are likely to be many factors that could contribute to the failure of MI holding for the DSM-IV criteria for MD. These include the specific wording of the diagnostic assessment instrument used (including effects of translation), interviewer qualifications and training, ascertainment procedures (e.g. in-patient, out-patient or population-based), the quality control processes implemented in the study, and the ethnicity of the sample.

In this report, we test the assumption that MD diagnosed using DSM-IV criteria in the CONVERGE sample is the same construct that is assessed by these same items in comparable Western countries, in particular the USA, England and The Netherlands. We assessed MI for the DSM-IV A criteria for MD in our CONVERGE Chinese sample of depressed women and four comparable samples from the USA and Europe matched on treatment and recurrence: the Depression Case Control Depression Network Study (DeCC-DeNT; Farmer *et al.* 2004; Cohen-Woods *et al.* 2009), the National Comorbidity Survey (NCS; Kessler *et al.* 1994), the National Epidemiologic Survey on Alcohol and Related Conditions (NESARC; Grant *et al.* 2003), and the Netherlands Study of Depression and Anxiety (NESDA; Penninx *et al.* 2008). For convenience, we refer to these as ‘Western’ samples.

As many factors could contribute to the failure of MI holding for the DSM-IV criteria for MD, we sought to test the hypothesis that the level of MI seen between the CONVERGE and the four Western samples would be qualitatively similar to that seen among comparable Western samples. We seek to distinguish between two possible outcomes. In one, CONVERGE is broadly typical of other samples in the observed level of inter-study MI. The second possible pattern of findings would be that MI is systematically poorer between CONVERGE and the Western studies than we see between the Western samples.

## Method

### Samples – CONVERGE

The analyses here reported were based on a total of 6008 cases of MD recruited as part of the CONVERGE study from 57 mental health centers and psychiatric departments of general medical hospitals in 45 cities in 23 provinces in China. The primary focus of CONVERGE was a molecular genetic study of MD. Given evidence that the genetic effects on MD are different in the sexes (Kendler *et al.* 2001), we collected only female participants who reported having four Han Chinese grandparents. Cases were excluded if they had a pre-existing history of bipolar disorder, psychosis or mental retardation. Cases were aged between 30 and 60 years, had ⩾2 episodes of MD meeting DSM-IV criteria (APA, 1994) with the first episode occurring between 14 and 50 years of age, and had not abused drugs or alcohol before their first depressive episode.

All subjects were interviewed using a computerized assessment system. Interviewers were postgraduate medical students, junior psychiatrists or senior nurses, trained by the CONVERGE team for a minimum of 1 week. The study protocol was approved centrally by the Ethical Review Board of Oxford University and the ethics committee in the participating hospitals in China.

The diagnosis of MD was established with the Composite International Diagnostic Interview (CIDI; WHO lifetime version 2.1, Chinese version), which utilized DSM-IV criteria (WHO, 1990). The interview was originally translated into Mandarin by a team of psychiatrists in Shanghai Mental Health Centre, with the translation reviewed and modified by members of the CONVERGE team.

### Western samples

Our goal was to create samples of women with MD from Western countries that would be maximally comparable to CONVERGE in terms of their clinical characteristics and their mode of assessment. We were able to obtain relevant samples from The Netherlands, UK, and USA.

For The Netherlands, the obtained sample was selected from NESDA and the Netherlands Twin Registry (NTR). NESDA is an ongoing longitudinal cohort study with a total of 2981 participants aged between 18 and 65 years. Samples with psychiatric illness and controls were recruited from the general population, general practices, and mental health clinics in The Netherlands (Penninx *et al.* 2008). Subjects with a primary clinical diagnosis of psychotic disorder, obsessive compulsive disorder, bipolar disorder, or severe addiction were excluded. The CIDI – lifetime version 2.1 (WHO, 1990) – was used to diagnose depressive and anxiety disorders according to DSM-IV algorithms (APA, 1994). Of the baseline samples, 1979 were female without a history of mania or psychosis. Applying the additional CONVERGE criteria including age (30–60 years), age of onset (14–50 years) and recurrence resulted in 407 cases remaining, among which 264 cases, the ones we examine in this study, reported treatment-seeking behavior. Treatment seeking was assessed based on the following available information: (i) current use of antidepressant; (ii) use of antidepressant in the last 3 years; (iii) recruited from secondary mental health care; or (iv) received psychotherapy/counselling in the last 6 months.

The NTR (Boomsma *et al.* 2006) was founded in 1987 and has ascertained large samples of twins and their relatives by questionnaires over the last 18 years. CIDI interview (version 2.1) based on DSM-IV was conducted twice in a subset of the NTR participants in 1997 and 2007, among which there were a total of 94 female samples in the NTR that met the diagnostic criteria for MD. After applying the CONVERGE entry criteria (i.e. age, age of onset, no history of mania or psychosis, no drug or alcohol dependence or alcohol dependence before the onset of depression, and treatment seeking for depression), a total of 30 cases remained for analysis. Both NESDA and NTR were based on the longitudinal study design from The Netherlands which were pooled together in a genome-wide association analysis – the Genetic Association Information Network (GAIN)-MDD study (Boomsma *et al.* 2008). Due to the sample compatibility and the fact that so few samples remained in the NTR cohort after applying the CONVERGE criteria, it was considered more appropriate to treat NESDA and NTR samples as one cohort (*n* = 294), hereafter termed NESDA for simplicity.

From the UK, we combined data from the DeCC and DeNT studies. The DeCC study recruited 1420 Caucasian individuals with recurrent unipolar depression from three clinical sites: London, Cardiff and Birmingham. Subjects were identified from psychiatric clinics, hospitals and general medical practices, and from volunteers responding to media advertisements. Subjects were recruited if they were aged >18 years and had experienced ⩾2 episodes of MD of at least moderate severity separated by at least 2 months of remission. All subjects were interviewed using the Schedules for Clinical Assessment in Neuropsychiatry (SCAN 2.1; WHO, 1993) which was designed to provide diagnoses according to both ICD-10 and DSM-IV criteria (APA, 1994). Subjects were excluded if they or a first-degree relative ever fulfilled criteria for mania, hypomania, schizophrenia, or experienced psychotic symptoms that were mood incongruent or present when there was no evidence of a mood disturbance. Other exclusion criteria were intravenous drug use with a lifetime diagnose of dependency or depression occurring solely in relation to alcohol or substance abuse, or depression only secondary to medical illness or medication, and a clear diagnosis of bipolar disorder, schizophrenia, schizoaffective disorder or acute or transient psychotic disorders in first- or second-degree relatives.

DeNt is a multicenter study designed for a genetic linkage analysis of unipolar depression in 470 Caucasian sibling pairs recruited from eight clinical sites in Europe and the USA. Probands were recruited if they fulfilled the DSM-IV or ICD-10 criteria for recurrent unipolar depression of moderate or severe degree and who had at least one similarly affected sibling. Clinical and psychological assessments were also based on SCAN 2.1 (WHO, 1993). Other exclusion criteria were the same as the DeCC. There were a total of 1505 female samples in both the DeCC and DeNT studies which has previously been combined for a GWAS study from the UK population and hence was used here as a single sample (Lewis *et al.* 2010). All cases were ascertained through clinical settings and there was no information on previous treatment for depression. Further filtering for CONVERGE entry criteria resulted in a total of 830 cases remaining.

Finally, two studies from the USA were selected. The NESARC is a longitudinal survey study sampled from the general US population and the subgroups of the population with its first wave of interview carried out in 2001–2002 (Grant *et al.* 2003). It is a representative sample of the non-institutionalized US population aged ⩾18 years. Depression was assessed using the Alcohol Use Disorder and Associated Disabilities Interview Schedule – IV (AUDADIS-IV) interview based on DSM-IV criteria (APA, 1994). There were a total of 42 093 baseline samples received from NESARC, of which 58% were female. After applying the CONVERGE criteria, a total of 629 samples remained for analysis.

The NCS is based on a stratified, multi-stage area probability sample of persons aged 15–54 years in the non-institutionalized civilian population in the 48 contiguous United States. Depression was assessed based on DSM-III-R (APA, 1994) using a modified version of the CIDI (WHO, 1990). There were a total of 8098 baseline samples received from the NCS, of which 53% were female. After applying the additional CONVERGE criteria, a total of 141 samples remained for analysis.

Our primary analyses in all the samples included in the study focused on the nine DSM-IV ‘A criteria’ for MD (APA, 1994) listed in Table 1. Our follow-up analysis of 14 symptoms disaggregated criterion A3 into four items (increased and decreased weight and increased and decreased appetite) and criteria A4 and A5 into two items each (insomnia and hypersomnia, and agitation and retardation, respectively).

**Table 1.** Age and percent endorsement rates for DSM-IV A criteria for major depression in the distinct samples

Features of the sample/criteria	CONVERGE (*N* = 6013)	DeCC-DeNT (*N* = 830)	NCS (*N* = 141)	NESARC (*N* = 629)	NESDA (*N* = 294)
Country	China	UK	USA	USA	The Netherlands
Structured interview	CIDI	SCAN	CIDI	AUDADIS	CIDI
Mean age, years (s.d.)	44.4 (8.9)	45.6 (8.4)	41.5 (6.8)	45.2 (8.6)	43.8 (8.0)
A1 – Depressed mood	99.3	99.4^a^	99.3	97.3	94.7
A2 – Loss of interest	98.7^a^	79.0	90.1	90.5	94.3
A3 – Weight/appetite change	90.0	82.5	93.6^a^	87.3	78.0
A4 – Sleep disturbance	94.9^a^	83.9	92.9	93.8	89.4
A5 – Psychomotor changes	90.2	95.2^a^	71.6	68.5	74.6
A6 – Fatigue	92.8	95.3^a^	91.5	90.3	93.9
A7 – Worthlessness	89.6	93.9^a^	75.2	78.4	86.7
A8 – Reduced concentration	97.0	91.7	89.4	94.1	97.7^a^
A9 – Suicidal ideation	75.8	67.7	84.4^a^	62.5	65.9
Mean endorsement	92.0^a^	87.6	87.6	84.7	86.1

a Highest endorsement rate for that A criterion.

CONVERGE, China, Oxford and Virginia Commonwealth University Experimental Research on Genetic Epidemiology; DeCC-DeNT, the Depression Case Control Depression Network Study (Cohen-Woods *et al.* 2009; Farmer *et al.* 2004); NCS, the National Comorbidity Survey (Kessler *et al.* 1994); NESARC, the National Epidemiologic Survey on Alcohol and Related Conditions (Grant *et al.* 2003); and NESDA; the Netherlands Study of Depression and Anxiety (Penninx *et al.* 2008); CIDI, Composite International Diagnostic Interview; SCAN, Schedules for Clinical Assessment in Neuropsychiatry; AUDADIS, Alcohol Use Disorder and Associated Disabilities Interview Schedule.

### Statistical methods

To evaluate the structural equivalence of the MD criteria A items in the CONVERGE sample with the four Western samples selected for similar clinical characteristics, a series of dimensional latent variable between sample MI analyses were conducted. MI testing was performed for both the nine and 14 disaggregated A MD criteria binary item sets. Initial exploratory model testing was carried out separately on each of the samples to determine the dimensionality of the two MD criteria sets in each sample. Determining an appropriate structural organization of the binary criteria sets is essential to minimize the impact on the non-invariance testing due to misspecified dimensionality.

Two key measurement parameters, factor loadings and thresholds, were examined to assess MI across samples. Factor loadings characterize the linear relationship between each of the binary MD items and the latent factor(s) that account for covariation among the items. These regressions are analogous to discrimination parameters in item response models. Thresholds indicate the location of where on the latent MD continuum each item optimally discriminates between lower and higher scores. Evidence for differential item functioning (DIF) here was restricted to assessing changes in global model fit indexes.

All model fitting and MI testing was performed with Mplus 7.11 software within a confirmatory factor analytic structure (Muthen & Muthen, 2012). The limited information weight least squares mean and variance adjusted robust estimator (WLSMV) was used for model optimization and fit. Three fit-indices were used to evaluate MI model comparisons. The Tucker–Lewis Index (TLI; Tucker & Lewis, 1973) and the Comparative Fit Index (CFI; Bentler, 1990) are relative fit indexes ranging between 0 and 1 with values ⩾0.95 considered to indicate good fitting models and values between 0.90 and 0.95 generally indicating adequate fits. The root mean square error of approximation (RMSEA; Steiger, 1990) was developed from the understanding that no model is an exact representation of data. Values of ⩽0.05 are considered to be good approximations.

All MI multiple group testing was carried out pairwise first between CONVERGE and each of the Western samples and then among the Western samples themselves. Our first analyses examined one factor models applied to the nine DSM-IV A criteria for MD. Our second set of analyses examined 14 criteria including disaggregated DSM criteria for sleep, weight, appetite and psychomotor changes. In our key test between the samples, we constrained criteria factor loadings and thresholds to be invariant across the two samples. Thus we were jointly testing metric (factor loadings) and scalar (thresholds) invariance across the samples. To avoid confounding the test of DIF with valid factor mean and variance differences, factor variance(s) and factor mean(s) in the second group were allowed to be free parameters. We then report the TLI, CFI and RMSEA values of this invariance model.

## Results

### Descriptive results

As seen in Table 1, the mean ages of the women at assessment was broadly similar in the five samples, all in their early to mid-40s. Table 1 also depicts the mean endorsement rates for the nine DSM-IV A criteria for the lifetime worst episode of MD in the five samples: CONVERGE, DeCC-DeNT, NCS, NESARC and NESDA. As expected, given the strict selection criteria, these rates are very high. While the average endorsement rates for these criteria were highest in the CONVERGE sample, the DeCC-DeNT sample had the individual highest endorsement rate for four of the criteria and CONVERGE for only two. At a symptomatic level, the samples are broadly similar with the CONVERGE study.

### Fitting between-sample models of measurement invariance to nine DSM criteria

We first fitted MI models to single factor solutions for the nine DSM-IV ‘A criteria’ for MD for the CONVERGE sample against each of the Western samples one at a time. These models imposing MI permitted the factor mean and variance to be estimated in the comparison samples while constraining both the factor loadings and thresholds to equality across samples. As seen in Table 2, the CFI and TLI results were similar – ranging from 0.86 to 0.92 and indicating a modestly acceptable, but not excellent fit of the model. The RMSEA, by contrast, were all under 0.03, indicating a good approximate model fit.

**Table 2.** Fit indices for measurement non-invariance for a one-factor solution applied to the nine DSM-IV criteria for major depression in the CONVERGE sample and four Western samples

Index	CONVERGE- DeCC-DeNT	CONVERGE-NCS	CONVERGE- NESARC	CONVERGE-NESDA	Mean	DeCC-DeNT-NCS	DeCC-DeNT NESARC	DeCC-DeNT-NESDA	NCS-NESARC	NCS-NESDA	NESARC-NESDA	Mean
CFI	0.896	0.916	0.888	0.884	0.896	0.946	0.820	0.809	0.908	0.916	0.741	0.856
TLI	0.877	0.877	0.868	0.863	0.871	0.936	0.788	0.774	0.892	0.900	0.694	0.831
RMSEA	0.028	0.025	0.028	0.029	0.028	0.030	0.038	0.050	0.032	0.049	0.049	0.041

CONVERGE, China, Oxford and Virginia Commonwealth University Experimental Research on Genetic Epidemiology; DeCC-DeNT, Depression Case Control Depression Network Study; NCS, National Comorbidity Survey; NESARC, National Epidemiologic Survey on Alcohol and Related Conditions; NESDA, Netherlands Study of Depression and Anxiety; CFI, Comparative Fit Index; TLI, Tucker–Lewis Index; RMSEA, root mean square error of approximation.

Two of the four Western studies – NCS and NESDA – used versions of the CIDI interview as did the CONVERGE study. If the nature of the interview (CIDI *v.* SCAN) played an important role in MI, we would have expected better fit indices for the CONVERGE-NCS and the CONVERGE-NESDA models than for the CONVERGE-DeCC-DeNT and the CONVERGE-NESARC. However, no such trend was seen.

Next, we fitted MI models for each combination of the Western sample pairs, and compared these model fits with those obtained when CONVERGE was paired with Western samples (Table 2). Here the results were more variable. From the perspective of the TFI and CFI, one of the model MI comparisons (DeCC-DeNT and NCS) fitted relatively well (TFI and CFI ~0.94), two moderately well (NCS-NESDA and NCS-NESARC with TFI and CFI ~0.90) and three relatively poorly (DeCC-DeNT-NESARC, DeCC-DeNT-NESDA, and NESARC-NESDA with TFI and CFI <0.83). The RMSEA was somewhat less discriminating with acceptable values for all models with the DeCC-DENT-NESDA having the worst and the DeCC-DeNT-NCS the best fit.

If the nature of the interviews were important in the between-sample MI, we would have expected the best fit to be between the two Western samples that both used the CIDI interview: the NCS and the NESDA. However, the best-fitting MI model was between two studies – DeCC-DeNT and NCS – that used different interviews, respectively the SCAN and the CIDI.

We then compared the mean values of the fit indices for the CONVERGE-Western and Western-Western models. As seen in Table 2, for all three indices, the mean values of the fit indices were higher for the CONVERGE-Western than for the Western-Western MI models.

### Fitting between-sample models of measurement invariance to 14 DSM criteria

We next carried out MI model testing for two factor solutions using the 14 disaggregated DSM-IV ‘A criteria’ for MD for the CONVERGE sample against each of the Western samples one at a time. We utilized the two-factor approach with the expectation that it would uncover typical and atypical depressive symptom factors. The DeCC-DeNT did not record these symptoms so we were left with only three Western samples for comparison. The three CONVERGE-Western MI models fit very similarly with CFI and TFI values ~0.90 and RMSEA values of ~0.04, all indicating a reasonable, but not excellent, fit (Table 3). Of the two Western-Western samples, the NESARC-NESDA fitted somewhat better by CFI and TLI (values ~0.91) compared to the NCS-NESDA (values ~0.87). Both models, however, had RMSEA values >0.05. The mean values of all three indices were lower for the CONVERGE-Western than for the Western-Western MI models.

**Table 3.** Fit indices for measurement non-invariance for a two factor solution applied to the fourteen disaggregated DSM-IV criteria for major depression in the CONVERGE sample and three Western samples

	CONVERGE-Western			Western-Western			
Index	CONVERGE-NCS	CONVERGE- NESARC	CONVERGE-NESDA	Mean	NCS-NESDA	NESARC-NESDA	Mean
CFI	0.903	0.906	0.904	0.904	0.882	0.918	0.900
TLI	0.899	0.894	0.891	0.895	0.866	0.907	0.887
RMSEA	0.038	0.043	0.038	0.040	0.064	0.062	0.063

CONVERGE, China, Oxford and Virginia Commonwealth University Experimental Research on Genetic Epidemiology; NCS, National Comorbidity Survey; NESARC, National Epidemiologic Survey on Alcohol and Related Conditions; NESDA, Netherlands Study of Depression and Anxiety.

As with the one-factor models, we saw no trend for the three samples that used the same CIDI interview – CONVERGE, NCS and NESDA – to have better fit MI models than comparisons with the samples using other interviews.

## Discussion

The goal of this report was to examine the degree to which DSM-IV criteria for MD perform similarly in patient assessments in China and Western countries. Our approach began by identifying four Western samples from the USA and Europe from which we could select individuals who met the entry criteria for the CONVERGE study: women above the age of 30 who sought treatment for recurrent MD with no history of bipolar illness. We then compared levels of MI using a dimensional latent variable approach across pairs of studies first for the nine DSM criteria for MD and then for the 14 disaggregated criteria.

The overall fit of the MI models varied from fair to relatively good. More importantly for our purpose, the global fits for the MI models were on average as good, or better, between CONVERGE and the Western samples as it was between the Western samples. Consistent with two prior relevant studies (Simon *et al.* 2002; Ryder *et al.* 2008), the DSM-IV criteria for MD appear to function in a similar way in Chinese women with recurrent MD as they do in comparable samples in the USA and Europe. This is true not only for a single-factor nine-criteria analysis but also with a two-factor 14-criteria analysis that captures the typical and atypical dimensions of depressive symptoms.

Our analyses permitted a rough determination of the importance of different structured interviews in the assessment of MD criteria across samples. In particular, we saw no evidence that MI models fitted better between the three samples that all used a version of the CIDI compared to samples that used other structured interviews. The particular interview that was utilized did not seem to have a major impact on the structure of the observed depressive criteria.

These findings suggest that despite substantial differences in ethnicity, culture and language, the underlying structure of the DSM criteria for MD is similar in East Asian, European and European-American women who meet DSM-IV diagnostic criteria for MD. That is, in these diverse populations, the MD criteria appear to define a latent variable of depression severity in a broadly equivalent manner.

A great deal has been written about the nature of MD in China and possible differences that may exist in its social contextualization from that seen in Western countries (Kleinman 1982, 2004; Lee 1999; Parker *et al*. 2001; Ryder *et al*. 2008). Far fewer reports have examined the more practical and empirically driven question we seek to address. Indeed, we could find only one prior study that specifically addressed this question. That study found the factor structure of nine DSM-IV criteria for MD across 15 cites, including China, to be very similar (Simon *et al.* 2002). One other report addressed relevant issues. Ryder *et al.* assessed somatic and psychological symptoms of depression in psychiatric out-patients in China and Canada (Ryder *et al.* 2008). Their psychometric analyses found no evidence for differential item functioning in either their psychological or somatic symptom subscale.

In a justly famous essay first published in 1923, Karl Birnbaum first developed the concept of pathogenic and pathoplastic features of psychiatric illness (Birnbaum, 1974). Birnbaum viewed pathogenic features as reflecting core etiologic processes which gave the disorder its specific character ‘its quality of being “thus and no other”’ (Birnbaum, 1974, p. 203). The pathoplastic features, according to Birnbaum, give content, coloring and contour to individual illnesses whose basic form and characters have already been biologically established’ (Birnbaum, 1974, p. 203]. In more modern terms, pathogenic features would reflect neurobiological processes while pathoplastic features would arise largely from cultural and psychological influences. For example, Birnbaum would likely argue that delusions in schizophrenia reflect pathogenic processes, but the content of the delusions typically arise from pathoplastic influences.

Our results are consistent with the hypothesis that the DSM-IV criteria for MD reflect largely pathogenic processes. We would have expected, given the wide cultural differences in our CONVERGE and Western samples, that if DSM-IV criteria for MD largely arose from pathoplastic influences, we would have seen much larger differences in their factorial structure across populations than we did. These results support the concept that MD, as defined by DSM criteria, is broadly defining depression in the ‘same way’ in China and in Western countries and should encourage efforts to study the wide range of possible risk factors for MD across diverse populations with the expectation that the results will prove to be broadly comparable. Our MI testing results suggest that this would be especially likely to be the case for genetic and biological risk factors that impact on the pathogenic processes that produce liability to MD.

Our results are of relevance for international efforts to localize genetic variants that predispose to MD. They suggest that, at least at the phenotypic level, patient samples derived from European and Chinese populations are likely to be broadly comparable. Our results do not, of course, provide any information about other issues in comparing genomic results across ethnicities including differences in allele frequencies and haplotype structure (Carlson *et al.* 2013).

### Limitations

These results should be interpreted in the context of six potential methodological limitations of the analyses present here. First, these results are only relevant to women with recurrent MD. It is an empirical question whether similar findings would arise from other samples or in the general population where many individuals would only be experiencing sub-clinical levels of depressive symptoms. Second, the CONVERGE sample was ascertained for recurrent MD in a clinical setting. As seen in Table 1, this produced very high endorsement rates for the DSM-IV criteria and similarly high levels were seen in our matched Western samples. Such high endorsement rates for binary items can introduce difficulties and instabilities in estimation and model fitting comparisons. Our results do not therefore necessarily apply to mild cases of MD that do not seek treatment and would commonly be seen in community samples. Third, analyses based on highly selected samples such as CONVERGE can lead to structural models that are not generalizable to other populations. It is important to note that the structures tested for MI here may not be representative of the MD A criteria structure in other samples especially the more mild cases typically seen in epidemiological samples. Fourth, given the more general nature of our question, our assessment of MI was limited to the global model fit indexes and we did not attempt to further clarify the sources of between sample misfits. Fifth, we were not able to control for the treatment status of the individuals when their symptoms were assessed during their lifetime worst depressive episode. It is possible that such treatments impacted on the symptom patterns reported. Finally, it was not possible to match exactly inclusion criteria across all studies. For example, only the DeCC excluded subjects based on a history of manic or psychotic symptoms in first-degree relatives. Such modest differences in sampling are unlikely, however, to have a major effect on the observed factor structure of criteria for MD.
